# Healing Beyond Words: A Randomized Controlled Trial of Sandplay and Art Therapies for PTSD Among Syrian Refugee Children in Jordan

**DOI:** 10.1155/ijpe/7636252

**Published:** 2026-06-10

**Authors:** Abdulqudos Al-Fakih, Abdallah Alzoubi, Ahmad Alrawashdeh, Jomana W. Alsulaiman, Saleh A. Ba-shammakh, Adi Khasawneh, Karin Dannecker, Khalid A. Kheirallah

**Affiliations:** ^1^ Department of Public Health and Family Medicine, Faculty of Medicine, Jordan University of Science and Technology, Irbid, Jordan, just.edu.jo; ^2^ Department of Pharmacology, Faculty of Medicine, Jordan University of Science and Technology, Irbid, Jordan, just.edu.jo; ^3^ Department of Applied Medical Sciences, Jordan University of Science and Technology, Irbid, Jordan, just.edu.jo; ^4^ Department of Pediatrics, Faculty of Medicine, Yarmouk University, Irbid, Jordan, yu.edu.jo; ^5^ Art Therapy Department, Weissensee School of Art (Kunsthochschule Berlin-Weißensee), Berlin, Germany

**Keywords:** children, conflict-related trauma, HTQ, PTSD, RCT, Syrian refugee

## Abstract

**Background:**

Expressive arts provide nonverbal therapeutic tools that can help children with post‐traumatic stress disorder (PTSD) overcome language and cultural barriers. This study compared the effectiveness of sandplay therapy and art therapy in reducing PTSD symptom severity among Syrian refugee children living in noncamp, host community settings in Jordan.

**Methodology:**

We conducted a three‐arm, parallel‐group, randomized controlled trial involving 102 Syrian refugee children (aged 5–13 years) with clinically diagnosed PTSD. Participants were randomly assigned to sandplay therapy (*n* = 32), art therapy (*n* = 42), or standard counseling (control, *n* = 28). Each group received one session per week for 12 weeks. The primary outcome was the total score on Part IV of the Arabic version of the Harvard Trauma Questionnaire (HTQ), assessed at baseline and postintervention. Between‐ and within‐group analyses, analysis of variance, and linear regression were used to evaluate changes in PTSD severity.

**Results:**

Baseline characteristics were comparable across groups, though severe PTSD was more prevalent in the sandplay group (40.6%), compared to the art therapy (11.9%) and control (25.0%) groups. After 12 weeks, sandplay therapy resulted in the greatest reduction in PTSD symptom severity (mean HTQ score difference: −22.4, *p* < 0.001), followed by art therapy (−10.8, *p* < 0.001) and standard counseling (−2.1, *p* = 0.002). Between‐group analyses confirmed sandplay′s superior efficacy over both art therapy and standard counseling (*p* < 0.001), whereas art therapy was significantly more effective than control.

**Conclusion:**

Both sandplay and art therapies significantly improved PTSD symptoms in conflict‐affected children, with sandplay demonstrating the greatest impact. These findings underscore the effectiveness of expressive, nonverbal interventions in addressing conflict‐related trauma among refugee populations and support their integration into comprehensive mental health programs.

**Trial Registration:**

ClinicalTrials.gov identifier: NCT06984198.

## 1. Introduction

Since the start of the conflict in Syria, approximately 6.5 million people have fled their country seeking refuge. In Jordan, around 650,000 Syrian refugees are officially registered with the United Nations High Commissioner for Refugees (UNHCR), with most of them (82%) are living within host (noncamp) community settings and about half are children [1, 2].

Previous research from Jordan demonstrated vulnerability of refugee children to mental health problems including post‐traumatic stress disorder (PTSD) and depression [3]. Consistent evidence of the potential psychological impact of exposure to conflict‐related trauma on refugee children exists especially from the Middle East region. Previous reports suggested that refugee children are particularly vulnerable to developing psychological problems, like PTSD, with more severity and at higher rates than adults [4, 5]. Additionally, postmigration and refugee status can negatively influence the mental health of children, as migrant and refugee children experience higher prevalence rates and higher severity of mental health problems compared to their counterparts from the host country [6, 7].

Children and adolescent refugees show a high prevalence of PTSD as indicated by a systematic review, which revealed a prevalence rate between 29% and 37% among refugee children residing in high‐income countries [8]. Similarly, Syrian refugee children residing in the Jordanian community have also demonstrated a high prevalence and severity of PTSD, with 31% of them experiencing moderate to severe symptoms [9]. Furthermore, the severity of PTSD among Syrian refugee children in Jordan was found to be influenced negatively by the experience of injury or loss of family members [3]. Previous research suggests that the prevalence and severity of PTSD symptoms tend to decrease with the time spent in the host country. However, among Syrian refugee children in Jordan, the prevalence of PTSD and emotional problems is still high [10].

The consequences of trauma in childhood are serious and need intervention as early as possible, considering that neglected childhood trauma may lead to chronic psychiatric conditions in adulthood. For instance, trauma in childhood is significantly associated with chronic depression in adulthood as 75% of chronically depressed patients reported multiple traumas in childhood [11].

In refugee settings, mental health providers often encounter challenging obstacles that hinder counseling and therapy such as language barriers, cultural differences, and reluctance to disclosure [12]. To address these barriers, other projective methods of therapy like art and sandplay have been introduced. Children express stress in different ways at various developmental stages, hence the need for these methods to provide a safe outlet for emotions, needs, and thoughts, and help integrate fragmented memories commonly observed in PTSD. Sandplay provides access to the unconscious, allowing individuals to express their inner thoughts and feelings in voluntary, cross‐cultural, and psychodynamic ways. This method is nonverbal and engages multiple senses, which makes it inherently therapeutic. Moreover, sandplay therapy can lower the threshold at which children start to engage in psychotherapy [13]. A meta‐analysis of 40 studies reported consistently positive results for sandplay therapy in various communities and across different practice settings [14].

Similarly, art therapy has demonstrated effectiveness in improving overall well‐being and reducing the severity of the PTSD symptomology, anxiety, depression, and other psychological problems among refugee and migrant children in postconflict settings across various cultures and communities [15, 16]. This projective method has been successfully used among Syrian refugee children. In a study that involved 64 Syrian refugee children residing in Turkey, art therapy was introduced in the form of music, movement, and drawing sessions over 5 days. The study used the Stressful Life Events (SLE) scale as pre and posttests, and showed that art therapy has effectively decreased the severity of PTSD, depression, and trait anxiety symptoms [17]. Another study conducted in the United States involving Syrian refugee children reported that art therapy did significantly decrease PTSD symptomology compared to control group [18]. Various psychotherapeutic interventions such as cognitive‐behavioral skills therapy, EMDR group therapy, art, dance, and movement therapy have been experimented with Syrian refugee children [19].

Although both methods have demonstrated therapeutic benefits, few studies have directly compared their effectiveness among refugee children. Comparing them can provide important evidence regarding which method may be more effective in alleviating PTSD symptoms. Moreover, comparing these interventions with conventional counseling can help identify practical, culturally adaptable options for low‐resource refugee settings. Systematic reviews indicate that few methodologically sound studies have examined art therapy in refugee children, and overall evidence remains limited, highlighting the need for comparative research [20].

Therefore, this study is aimed at estimating and comparing the effectiveness of sandplay and art therapies in alleviating PTSD symptomology among Syrian refugee children living in noncamp (host community) settings in Jordan. Additionally, the study compared the effectiveness of these two therapeutic methods to that of conventional counseling.

## 2. Methodology

### 2.1. Study Design

This was a three‐arm, parallel‐group, nonblinded randomized controlled trial (RCT) designed to compare the effectiveness of sandplay therapy, art therapy, and standard counseling in mitigating PTSD symptoms among Syrian refugee children over a span of 12 weeks.

### 2.2. Participants

A total of 129 Syrian refugee children, ages 5–13, referred from a local NGO database of Syrian refugees living in Amman, Jordan, and actively seeking mental health services were recruited to participate in the study. Participants were randomly assigned into one of the three experimental groups: control, art, or sandplay therapy. Upon gaining parent or legal guardian permission, children qualified for participation after confirmation of the diagnosis of PTSD. Each patient was interviewed twice, first by a psychologist using the Harvard Trauma Questionnaire (HTQ) Part VI, and immediately after by a psychiatrist employing the structured clinical interview for DSM‐IV Axis I disorders [21–23]. Thereafter, participating children were subjected to a defined set of inclusion criteria to the study, as follows: (1) reporting trauma related to experiences associated with being in a combat region in their home country (e.g., witnessed death, shrapnel injuries, and/or any forms of war violence); (2) had not participated in counseling prior or during the study; (3) did not have preexisting psychosocial issues; and (4) were not previously diagnosed with psychotic and cognitive deficit disorders were included in the study. Children that did not meet the inclusion criteria and/or did not reach the HTQ cut‐off mark of 2.0 (equivalent to a minimum score of 32) were not included in the study, and the parents or legal guardians of the children were given referral information if they wished their children to participate in counseling elsewhere.

### 2.3. Interventions

#### 2.3.1. Sandplay Therapy

The objects involved in this form of therapy were: sand tray, sand, and multiple figurines in all categories (people, animals, buildings, vehicles, vegetation, structures, natural objects, and symbolic objects). A wooden sand tray (75 × 50 × 7 cm), half filled with sand, was placed on a table that can be easily accessed by the child (waist‐high). The child was provided with water, tools (e.g., shovel, sieve, tubes), and miniature objects that are organized in the following categories: living objects, scenery, transport, equipment, people, animals, buildings, vegetation, structures, natural objects, and symbolic objects. The first stage involved construction of the “sandworld,” where the child was instructed by the psychologist to build a sand picture with the present miniatures. Sessions were 30–40 min long and were done in silence. No judgment or comment was made from the psychologist or research assistant present, and instead they recorded each subject′s layout and placement of figurines during every session on a form. The second stage of this process commenced following the subject′s creation of the sand picture where the child, if comfortable, chose to share the story or narrative about the sand picture they created. This allowed the subject to clarify personal meanings related to their sand picture and/or integrate new feelings and insights that may have emerged through the creation of the sand picture.

#### 2.3.2. Art Therapy

The instruments used in this form of therapy were: a blank 8″ × 11″ paper and coloring utensils (markers, crayons, and color pencils). During each 30‐min session, the child was informed to draw whatever they feel with the coloring utensils provided. Possible drawing topics may include: their family, a memory of their life back in their country of origin, or something from their imagination. Each subject′s completed drawing was then collected, photographed, and documented following every session. This approach is a modification of art therapy since it was mainly based on creation of a drawing upon an instruction, not including a verbal exploration of the drawings. The procedure was adapted upon supervision of an art therapist.

Individuals from both intervention groups did not demonstrate significant improvement from either therapy participated in individualized support or therapy as recommended by the clinical psychiatrist. Strategic interventions (group therapy, play therapy, music therapy, individual therapy, etc.) continued until significant improvement was observed.

Therapy Sessions:

In both art and sand groups, each child underwent 14 sessions, once per week, for a duration of 14 weeks. The first and last sessions served as pre and postassessment sessions, respectively. The remaining 12 weeks were considered the intervention period, where depending on the child′s randomized group assignment, they underwent either no formal (control), art, or sandplay therapy.

#### 2.3.3. Session 1

The research team established a safe environment for the child, obtained the child′s background history and consent for participation in the study, and took a preassessment of the child′s condition to determine the severity of their PTSD as established by their answers provided on their translated HTQ.

#### 2.3.4. Sessions 2–13

Children in the sandplay and art therapies intervention groups participated in their assigned therapies once a week. The therapist present during each session followed the guidelines outlined in the manual of each form of therapy. Expected duration of each session was 1 h, where 15 min served as an opportunity to engage in an optional verbal explanation of their creation. The children were given the option to not participate in each week′s session. Each child′s sandtray or art pieces were photographed for documentation and analysis purposes.

#### 2.3.5. Session 14

The last session served as a closing meeting for the children′s involvement in the study. Postassessment was taken using the same questionnaire used at the beginning of the study.

#### 2.3.6. Location

Zad Children Center in Amman, Jordan, was chosen to conduct the current study. Since January 2014, Zad Children Center, a nongovernmental organization, has provided holistic support to Syrian children and their families with psychosocial issues.

#### 2.3.7. Interventionists

Zad Children Center employed a qualified group of therapists, psychosocial support specialists, and a board‐certified psychiatrist who designed and supervised the interventions listed in the current study.

### 2.4. Outcome Measurement

The primary outcome was the total score on Part IV of the HTQ, Arabic version. This section comprises 16 PTSD‐related items, each rated from 1 (“not at all”) to 4 (“extremely”), yielding a total possible score ranging from 16 to 64. Higher total scores indicate greater PTSD severity. The HTQ was administered at baseline and again after the 12th session to capture changes in symptom severity over time. Children with higher baseline scores of 40 or above were classified as having severe PTSD, whereas those with lower totals were categorized as mild (score of 16) or moderate (scores between 17 and 39), following established scoring benchmarks in similar conflict‐affected populations.

The same clinicians and research assistants who administered these measures at baseline repeated them at the study′s conclusion, allowing for a direct comparison of pre and postintervention scores.

### 2.5. Statistical Analysis

All analyses were performed using Stata (StataCorp, College Station, Texas, United States, Version 16). Baseline characteristics were summarized as means (± standard deviations (SD)) for continuous variables and as frequencies (percentages) for categorical variables. Differences in baseline variables (e.g., age, sex, PTSD severity) across the three intervention arms were tested using one‐way analysis of variance (ANOVA) for continuous measures and chi‐square tests for categorical measures.

Paired *t*‐tests evaluated within‐group changes from baseline to postintervention. The mean difference and standard error were calculated for each arm, and percentage changes were also computed to illustrate the relative magnitude of improvement. Between‐group differences in these prepost changes were then assessed using one‐way ANOVA, with post hoc tests (i.e., Tukey) for pairwise comparisons. Minor deviations from normality were considered acceptable due to the robustness of ANOVA with moderate sample sizes.

Univariable and multivariable linear regression models were employed to compare the effectiveness of the three interventions on mean difference of HTQ scores. Multivariable models controlled for age, sex, and baseline PTSD severity (mild, moderate, or severe). Regression coefficients (*β*) and 95% confidence intervals (95% CI) were reported to quantify the magnitude and statistical significance of treatment effects. A two‐sided *p* value of less than 0.05 was considered statistically significant for all analyses.

### 2.6. Ethical Considerations

Ethical approval for this study was obtained from the Institutional Review Board (IRB) of King Abdulla University Hospital and Jordan University of Science and Technology (No. 464‐2015). With thorough information provided about potential risks, benefits, and the option to withdraw from the study at any time. Parents and guardians willingly consented, and children voiced their assent. Confidentiality of participant data was strictly maintained, and no personal identifiers were used in any analytic dataset or reporting. The trial adhered to the ethical principles outlined in the Declaration of Helsinki.

## 3. Results

Out of the total 200 participants screened for eligibility, 150 were invited to participate. Of those, 129 participants provided parental informed consent and were initially randomized. Only 102 participants completed the study. Of these, 42 in art therapy, 32 in sandplay therapy, and 28 in standard counseling groups. Baseline demographics and clinical characteristics (Table 1) showed a broadly similar distribution in terms of age and sex, with a mean age of 9.0 years (SD ± 1.6). Nonetheless, sandplay therapy included a higher number of children presenting with severe PTSD (40.6%) compared to the art (11.9%) and control groups (25.0%), whereas mild PTSD occurred more frequently in the art (33.3%) and control (28.6%) arms (*p* = 0.046)(Table 1).

**Table 1:** Distribution of the baseline characteristics across the treatment groups.

	Total *n* = 102	Intervention group (therapy)			*p*
		Art *n* = 42	Sandplay *n* = 32	Control *n* = 28	
Age, mean (SD)	9.0 (1.6)	9.0 (1.5)	8.9 (1.6)	9.0 (2.2)	0.973
Age groups, *n* (%)					0.352
School age (5–9)	62 (60.8)	29 (69.1)	18 (56.3)	15 (53.6)	
Preadolescent 10–13	40 (39.2)	13 (30.9)	14 (43.8)	13 (46.4)	
Gender, *n* (%)					0.353
Girls	49 (48.0)	22 (52.4)	12 (37.5)	15 (53.6)	
Boys	53 (52.0)	20 (47.6)	20 (62.5)	13 (46.4)	
PSTD severity, *n* (%)					0.046
Mild	26 (25.5)	14 (33.3)	4 (12.5)	8 (28.6)	0.036
Moderate	51 (50.0)	23 (54.8)	15 (46.9)	13 (46.4)	0.884
Severe	25 (24.5)	5 (11.9)	13 (40.6)	7 (25.0)	0.017

Statistical tests indicated that the HTQ data were slightly nonnormally distributed, with significant results on both Kolmogorov–Smirnov (*p* = 0.006) and Shapiro–Wilk (*p* = 0.001) tests. However, the distribution of the data was deemed acceptable based on skewness and kurtosis values: preintervention HTQ scores had skewness = 0.365 and kurtosis = −0.889, whereas postintervention scores had skewness = 0.593 and kurtosis = −0.413.

Parametric tests were applied despite nonnormality because skewness and kurtosis were within acceptable limits, the sample size was moderate (supporting the central limit theorem), and no extreme outliers were present. Nonparametric analysis confirmed consistent results.

### 3.1. Changes in HTQ Scores Within and Between Groups

As shown in Table 2, all three intervention groups were associated with statistically significant decreases in total HTQ scores from baseline to Week 12. Sandplay therapy demonstrated the largest absolute reduction (mean difference: −22.4, SE: 9.1, *p* < 0.001), followed by art therapy (mean difference: −10.8, SE:5.6, *p* < 0.001). The control group showed a smaller, yet significant, reduction (mean difference: −2.1, SE: 3.2, *p* = 0.002). Percentage reductions in total HTQ scores were −50.7% in sandplay, −28.2% in art, and −5.2% in the control group.

**Table 2:** Differences in the HTQ score between baseline and postintervention for the treatment arms.

Treatment group	Number	Mean HTQ scores (SD)		Mean difference (SE)	Percentage change (%)	*p*
		Pre	Post			
Art therapy	42	38.3 (8.3)	27.5 (8.4)	−10.8 (5.6)	−28.2	< 0.001
Sandplay therapy	32	44.3 (11.1)	21.8 (4.9)	−22.4 (9.1)	−50.7	< 0.001
Control	28	40.2 (9.5)	38.1 (8.3)	−2.1 (3.2)	−5.2	0.002

**Figure 1:**
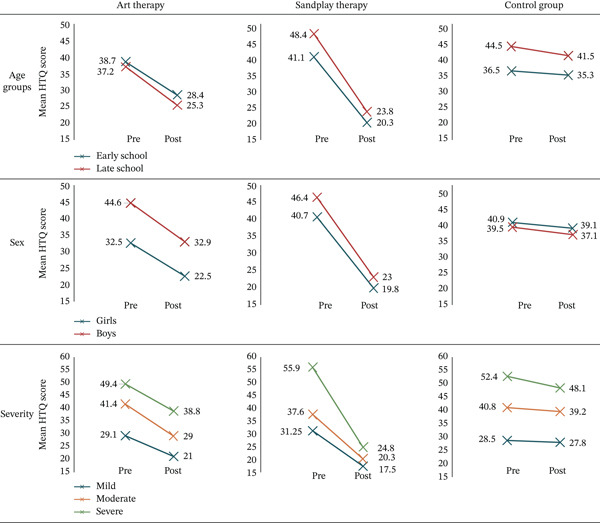
Subgroup analysis by age groups, sex and PSTD severity by the treatment group.

Figure 1 illustrates that both art and sandplay therapies foster significant reductions in PTSD symptoms across age groups, sexes, and severity levels, with sandplay showing the steepest declines in HTQ scores, especially among children with severe PTSD at baseline. By contrast, the control group exhibits smaller reductions overall, indicating that standard counseling still offers some relief.

The HTQ demonstrated high reliability, with Cronbach′s alpha of 0.931 at baseline and 0.937 postintervention, indicating excellent internal consistency.

Moreover, a one‐way ANOVA was conducted to compare the effectiveness of the interventions on PTSD symptoms using the HTQ. There was a significant effect of intervention type on HTQ scores, *p* < 0.001, *η* ^2^ = 0.43, indicating a large effect size.

Post hoc comparisons (Tucky) revealed the following mean differences:

• Art therapy vs. counseling: Art therapy showed significantly lower PTSD scores (MD = −10.667, *p* < 0.001).
• Sandplay therapy vs. counseling: Sandplay therapy also resulted in significantly lower scores (MD = −16.330, *p* < 0.001).
• Sandplay therapy vs. art therapy: Sandplay therapy also resulted in significantly lower scores (MD = −5.664, *p* = 0.005).

### 3.2. Group Effects and Baseline Predictors

Table 3 displays unadjusted and adjusted mean differences in total HTQ scores, emphasizing each treatment arm′s impact relative to the control group, as well as potential moderating effects of age, sex, and baseline PTSD severity. Unadjusted models indicated that art therapy reduced total HTQ scores by an average of 8.7 points (95% CI: 5.6, 11.8; *p* < 0.001) more than standard counseling, whereas sandplay therapy showed an even greater reduction of 20.4 points (95% CI: 17.0, 23.7; *p* < 0.001). These results remained consistent after adjusting for age, sex, and PTSD severity.

**Table 3:** Unadjusted and adjusted mean difference in the HTQ score of the study arms and variables.

	Univariable regression		Multivariable regression	
	Coeff (95% CI)	*p*	Coeff (95% CI)	*p*
Groups				
Control (ref)	1	*—*	1	*—*
Art	**8.7 (5.6, 11.8)**	**< 0.001**	**9.7 (6.9, 12.5)**	**< 0.001**
Sand	**20.4 (17.0, 23.7)**	**< 0.001**	**18.9 (15.9, 21.9)**	**< 0.001**
Age in years	0.4 (‐0.7, 1.6)	0.483	0.3 (−0.4, 0.9)	0.435
Female sex	−3.7 (−7.6, 0.3)	0.068	−0.3 (−2.6, 2)	0.786
PSTD severity at baseline				
Mild	**−4.4 (−8.8, −0.1)**	**0.046**	−2.5 (−5.3, 0.2)	0.071
Moderate	1	—	1	—
Severe	**8.3 (3.9, 12.8)**	**< 0.001**	**6.4 (3.5, 9.3)**	**< 0.001**

Baseline PTSD severity exerted a significant effect on the overall change in HTQ scores. Severe PTSD at baseline was associated with a higher total score reduction compared to moderate severity (*β* = 8.3 unadjusted; *β* = 6.4 adjusted; *p* < 0.001), whereas mild PTSD showed a smaller decrease in unadjusted models (*β* = −4.4, *p* = 0.046), which was attenuated in the adjusted models (*p* = 0.071). Neither age nor sex emerged as statistically significant predictors.

## 4. Discussion

This study examined the effectiveness of sandplay and art therapies in alleviating PTSD symptoms among Syrian refugee children living in noncamp, host community settings in Jordan. The findings indicate that both therapies were effective in reducing PTSD symptoms, with sandplay therapy exhibiting superior efficacy compared to art therapy and standard counseling. These findings underscore the importance of nonverbal therapeutic approaches in addressing psychological trauma in refugee children, particularly in resource‐limited settings where access to specialized mental health care is constrained.

A more nuanced distinction between art and sandplay therapy may help explain the observed differences in outcomes. Sandplay therapy involves tactile interaction with sand and miniature objects, which may engage interoceptive and proprioceptive sensory systems and function partly as a somatic‐sensory intervention supporting emotional regulation and trauma processing [24]. In contrast, art therapy relies more on symbolic and expressive processes, allowing children to externalize traumatic experiences through creative activities such as drawing or painting. These differing mechanisms may contribute to the stronger symptom reductions observed in the sandplay therapy group, although both interventions demonstrated meaningful therapeutic benefits [25].

Children with PTSD are particularly vulnerable to retraumatization during traditional, verbal‐based therapies that require direct discussion of traumatic experiences. Nonverbal approaches, such as sandplay and art therapies, provide alternative pathways for emotional expression, allowing children to process trauma through symbolic representation rather than verbal confrontation [13]. These methods create a safer therapeutic space, facilitating emotional healing while reducing the risk of distress, and bypassing language barriers and cultural differences that often hinder traditional verbal counseling. This is particularly relevant in multicultural settings, like Jordan, where refugees come from diverse linguistic and cultural backgrounds.

Both therapies require basic materials such as sand, miniatures, paper, and coloring utensils which makes them cost‐effective options, particularly in low‐resource environments where mental health services are limited; however, adequate training before implementation is necessary. Community centers, schools, or even makeshift clinics could be adapted to provide these therapeutic interventions, expanding access to psychological support for refugee children without requiring extensive infrastructure or highly specialized personnel.

Our findings align with previous research demonstrating the benefits of sandplay therapy for children exposed to war, natural disasters, and other forms of trauma. For instance, studies from Nepal and Uganda illustrated the effectiveness of sandplay therapy in lowering the severity of PTSD symptoms among street children or those living in a shelter and having a history of physical abuse or trauma [26–28]. Moreover, an RCT was conducted among school‐age children suffering from chronic conditions showed that sandplay therapy was more effective than standard counseling in lowering the levels of anxiety, depression, withdrawal, and social behavioral problems. Further, schoolchildren who developed PTSD as a result of cyberbullying responded significantly better to sandplay therapy than their counterparts in the control group [29, 30].

The efficacy of sandplay therapy in stimulating the brain‐psyche connection through symbolic play may explain its superior outcomes compared to both art therapy and standard counseling. Trauma is often encoded as imagery in memory, and nonverbal expressive therapies provide a direct pathway for processing and integrating these memories [13, 31]. Additionally, prior research suggests that sandplay therapy can improve adherence to other therapeutic interventions, making it a valuable complementary tool in PTSD treatment [32].

Our study also found that art therapy was significantly more effective than standard counseling in alleviating PTSD symptoms, supporting existing literature on its benefits for trauma‐exposed children [15–18]. For instance, a controlled interventional study conducted among unaccompanied asylum‐seeking children found that a 5‐week expressive arts program resulted in more positive outcomes in PTSD symptoms and life satisfaction among the expressive arts group compared to a control group [33]. However, some reports have questioned the strength of evidence supporting art therapy for PTSD. A systematic review of studies published between 2010 and 2020 suggested that although art therapy shows promise, many studies lacked control groups or involved art therapy as part of broader expressive or group therapy programs [34]. Notably, our study assessed art therapy as a standalone intervention and included a direct comparison with a control group, strengthening the validity of our findings. Despite these promising results, further research is needed to explore the long‐term benefits of art therapy, refine standardized intervention protocols, and assess its applicability across different refugee populations [35].

We acknowledge that differences in baseline PTSD severity across the three groups, along with unequal group sizes due to attrition, may have partially influenced postintervention outcomes through regression to the mean. Nevertheless, analyses adjusted for baseline severity confirmed that both art and sandplay therapies produced significant reductions in HTQ scores compared with controls, indicating a meaningful treatment effect beyond baseline differences.

### 4.1. Clinical Implications

The findings suggest that both art and sandplay therapies are promising approaches for reducing PTSD symptoms among refugee children. These nonverbal, expressive interventions may be particularly suitable in refugee settings, where language barriers and difficulties in verbalizing traumatic experiences can limit the effectiveness of conventional counseling. Although sandplay therapy showed greater reductions in PTSD symptoms, both therapies offer developmentally appropriate and culturally adaptable methods for trauma processing. In resource‐limited refugee‐hosting settings such as in Jordan, integrating these creative‐ and play‐based therapies into community‐ and school‐based psychosocial programs, and training healthcare professionals, social workers, and community practitioners in their use, may help expand access to trauma‐informed care and reduce the long‐term psychological impact of war‐related trauma among displaced children.

### 4.2. Limitations

Although our study provides significant insights into the effectiveness of sandplay and art therapies for Syrian refugee children, certain limitations should be acknowledged. First, the study population was limited to Syrian refugee children in Jordan, which may affect the generalizability of the results to other refugee groups. Second, the study focused solely on PTSD symptoms without assessing coexisting conditions such as anxiety, depression, or personality disorders. Future research should explore the broader mental health impact of these therapies and investigate their long‐term efficacy. Conducting larger‐scale studies with diverse populations with adoption of mixed‐method design involving qualitative analysis could lead to establishing standardized protocols for the implementation of these therapies. Third, due to the nature of the interventions, blinding was not feasible, as participants, therapists, and assessors were aware of the treatment allocation. This is particularly relevant since the primary outcome measure, HTQ, was administered via interviews. One notable limitation is the loss to follow‐up, which occurred in 21% of the participants (27 out of 129 initially randomized). Assessing the reasons for this loss was challenging due to the vulnerable nature of the population and the transient living conditions commonly experienced by refugees. Potential reasons for dropout may include temporary living conditions, lack of economic means for transportation, and competing family priorities. These factors can make it difficult to maintain consistent engagement with participants over the course of the study.

## 5. Conclusion

The demonstrated effectiveness of sandplay and art therapies in alleviating PTSD symptoms among Syrian refugee children in Jordan highlights their potential as valuable tools for mental health support in resource‐limited settings. By prioritizing these therapies, Jordan can enhance its capacity to address the psychological needs of vulnerable refugee populations, promote their well‐being, and foster their successful integration into host communities. Investing in the training of local providers and integrating these therapies into public health initiatives will be crucial steps toward achieving these goals.

## Author Contributions

Abdulqudos Al‐Fakih and Abdallah Alzoubi: formal analysis, data collection, writing original manuscript, and review and editing. Ahmad Alrawashdeh, Jomana W. Alsulaiman, Saleh A. Ba‐shammakh, and Adi Khasawneh: methodology, writing original manuscript, and review and editing. Karin Dannecker: conceptualization, methodology, formal analysis, writing original manuscript, and review and editing. Khalid Kheirallah: conceptualization, methodology, formal analysis, writing original manuscript, review and editing, and supervision. Abdulqudos Al‐Fakih and Abdallah Alzoubi contributed equally to this work.

## Funding

This study was supported by the Jordan University of Science and Technology (10.13039/501100004035) (20160004).

## Conflicts of Interest

The authors declare no conflicts of interest.

## Data Availability

The data that support the findings of this study are available from the corresponding author upon reasonable request.
